# Synthesis, Characterization, Antibacterial and Antifungal Evaluation of Novel Monosaccharide Esters

**DOI:** 10.3390/molecules17078661

**Published:** 2012-07-23

**Authors:** Yi Shen, Yufeng Sun, Zhipei Sang, Chengjun Sun, Ya Dai, Yong Deng

**Affiliations:** 1 West China School of Public Health, Sichuan University, Chengdu 610041, China; 2 Harmful Components and Tar Reduction in Cigarette, Sichuan Key Laboratory. No. 56, Section 1, Chenglong Road, Chengdu 610066, China; 3 Key Laboratory of Drug Targeting and Drug Delivery System of the Education Ministry, Department of Medicinal Chemistry, West China School of Pharmacy, Sichuan University, Chengdu 610041, China

**Keywords:** carbohydrate-acetonides, 3-(2-furyl)acrylate monosaccharide esters, menthyloxycarbonyl monosaccharide esters, antibacterial activities, antifungal activities

## Abstract

A novel series of 3-(2-furyl)acrylate monosaccharide esters **Ia**–**f** and menthyloxycarbonyl monosaccharide esters **IIa**–**f** were designed and synthesized. The chemical structures of the target compounds were confirmed by IR, ^1^H- and ^13^C-NMR and ESI-MS, and the target compounds were investigated for their *in vitro* antibacterial and antifungal activities. The antibacterial screening results showed that the 3-(2-furyl)acrylate monosaccharide ester derivatives **Ia**–**f** were either inactive or only weakly active against the three Gram-positive bacterial strains tested, whereas the menthyloxycarbonyl monosaccharide ester derivatives **IIa**–**f** exhibited higher levels of activity, with compound **IIe** being especially potent. The results of the antifungal screening revealed that compounds **Ib**, **Ie**, **IIb** and **IIc** displayed potent *in vitro* activities, whereas **If** and **IIf** showed promising activities against all of the microorganisms tested, with **If** exhibiting levels of activity deserving of further investigation.

## 1. Introduction

Microbial food contamination problems have been the cause of much public concern over the last few decades because of an increase in the number of infections and diseases originating from the consumption of spoiled food [1]. Antibacterial and antifungal agents are necessary for food preservation, especially for food processors, because bacterial and fungal growth are important causes of food spoilage. For this reason, many investigators have focused their research efforts on finding new efficient, low toxicity and environmentally friendly antibacterial and antifungal agents.

Sugar esters have been widely used as cosmetic and pharmaceutical industries for many years because they are considered biocompatible, biodegradable, and nontoxic and can be synthesized from renewable resources [2,3,4,5,6,7]. Furthermore, sugar esters have attracted considerable research interest in recent years because they have exhibited a variety of biological activities, including insecticidal [8], antitumor [9,10,11,12,13], antimicrobial and antifungal properties [14,15,16,17,18]. These results prompted us to design and synthesize a novel series of sugar esters in the hope that we might find some promising antimicrobial or antifungal agents. From a review of the literature, there have been many reports concerning propyl 3-(2-furanyl)acrylate ester and (−)-menthol and their applications in the food, beverage and cosmetics industries. Furthermore, 3-(2-furyl)acrylic acid and its ester derivatives and (−)-menthol have also been reported as antimicrobial agents [19,20,21,22,23]. But these compounds have poor solubility in water and thus result in low bioactivity. Inspired by these observations, we planned to couple 3-(2-furyl) acrylic acid or (−)-menthol with monosaccharides to obtain the corresponding water-soluble monosaccharide esters, as it was envisaged that these novel compounds would combine the favorable properties of sugars with either the 3-(2-furyl)acrylic acid esters or (−)-menthol and will be more bioavailable.

**Scheme 1:**
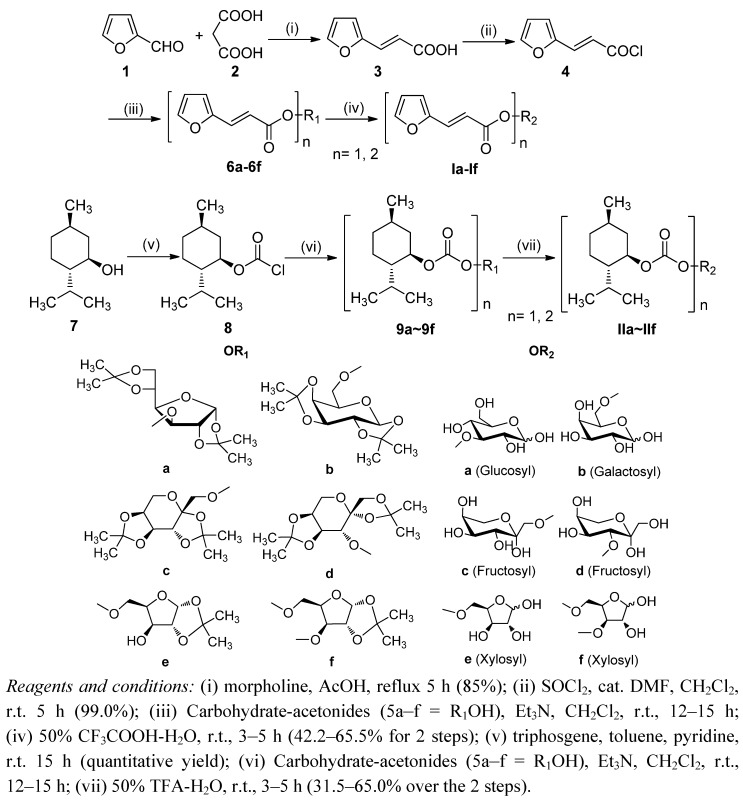
Synthesis of compounds **Ia**–**f** and **IIa**–**f**.

Based upon the aforementioned considerations, herein we describe the synthesis of two novel series, including a series of 3-(2-furyl) acrylate monosaccharide esters **Ia**–**f** and a series of menthyloxycarbonyl monosaccharide esters **IIa**–**f** (Scheme 1). 

Furthermore, their *in vitro* antibacterial activities against *Bacillus subtilis*, *Staphylococcus aureus*, *Staphylococcus* *epidermidis*, *Escherichia coli* and *Pseudomonas aeruginosa*, and antifungal activities against *Aspergillus flavus*, *Aspergillus* *niger*, *Aspergillus fumigatus* and *Geotrichum candidum* were investigated.

## 2. Results and Discussion

### 2.1. Chemical Synthesis

The target compounds **Ia**–**f** and **IIa**–**f** were synthesized according to conventional procedures as outlined in Scheme 1. Compound **3** [24] was prepared by a modified procedure which provided the product more conveniently and in higher yield, and was subsequently reacted with thionyl chloride to afford α-furanacryloyl chloride **4**. Compounds **6a**–**f** were obtained via the esterification of intermediate **4** with the corresponding one anomer of *O*-isopropylidene-protected monosaccharides **5a**–**f** in dry dichloromethane in the presence of Et_3_N at room temperature. Subsequent deprotection of the *O*-isopropylidenes with a 50% TFA-H_2_O solution at room temperature gave the desired 3-(2-furyl)acrylate monosaccharide esters **Ia**–**f** as a mixture of anomers for **Ia**, **Ib**, **Ie** and **If** due to mutarotation [25]. The menthyloxycarbonyl monosaccharide esters **IIa**–**f** were synthesized from the readily available alcohol (−)-menthol (**7**), which was reacted with triphosgene and pyridine in toluene to give menthyl chloroformate **8** in quantitative yield [26]. Chloroformate **8** was taken into the next step without purification and reacted with the one anomer of *O*-isopropylidene protected monosaccharides **5a**–**f** in dry dichloromethane in the presence of Et_3_N at room temperature to afford the corresponding menthol carbonates **9a**–**f** in high yields. Subsequent deprotection of the *O*-isopropylidenes with a 50% TFA-H_2_O solution at room temperature gave the desired anomeric mixture of menthyloxycarbonyl monosaccharide esters **IIa**–**f**. Among them, **IIa**, **IIb**, **IIe** and **IIf** were obtained as a mixture of anomers due to mutarotation. All of the target compounds were purified by silica gel flash column chromatography, and their chemical structures were confirmed by IR, ^1^H- and ^13^C-NMR and ESI-MS. To the best of our knowledge, none of these monosaccharide esters have been reported in the literature, and therefore represent novel compounds.

### 2.2. Antibacterial and Antifungal Activities

#### 2.2.1. Antibacterial Activities

The *in vitro* antibacterial activities of the monosaccharide esters **Ia**–**f** and **IIa**–**f** were tested against five bacterial strains, including the three Gram-positive organisms, *B. subtilis*, *S. aureus*, and *S.* *epidermidis*, and the two Gram-negative organisms *E. coli* and *P. aeruginosa*. The antibacterial assays were conducted according to the NCCLS (National Committee for Clinical Laboratory Standards) document M100-S12 method [27]. Standard antibacterial agents, including penicillin and streptomycin, were also screened under identical conditions for the sake of comparison. The minimum inhibitory concentrations (MIC) values of the tested compounds are shown in Table 1.

**Table 1:** MIC values (µg/mL) of the tested compounds against selected bacterial strains.

Compound	MIC (µg/mL)				
	Gram-positive			Gram-negative	
	*B*. *subtilis*	*S*. *aureus*	*S. epidermidis*	*E*. *coli*	*P*. *aeruginosa*
**Ia**	32	32	32	>64	>64
**Ib**	32	32	32	>64	>64
**Ic**	>64	32	>64	>64	>64
**Id**	>64	32	>64	>64	>64
**Ie**	32	32	>64	>64	>64
**If**	32	>64	>64	>64	>64
**IIa**	32	16	32	>64	>64
**IIb**	8	32	32	>64	>64
**IIc**	32	32	16	>64	>64
**IId**	32	32	32	>64	>64
**IIe**	2	2	8	>64	>64
**IIf**	8	16	16	>64	>64
**3**	8	32	16	>64	>64
**(−)-Menthol**	8	16	8	8	4
**Penicillin**	1	1	4	32	16
**Streptomycin**	8	8	16	4	2

Negative control 5% DMSO---no activity.

The compounds tested clearly exhibited varying degrees of antibacterial activity. The 3-(2-furyl)acrylate monosaccharide ester derivatives **Ia**–**f** were either inactive or only weakly active against the three Gram-positive bacterial strains tested, whereas the menthyloxycarbonyl monosaccharide ester derivatives **IIa**–**f** showed greater levels of activity, with compound **IIe** exhibiting remarkably high levels of antibacterial activities against all three bacterial strains. It was also shown that all of the target compounds were inactive against the gram-negative bacterial strains tested.

#### 2.2.2. Antifungal Activities

The *in vitro* antifungal activities of the monosaccharide esters **Ia**–**f** and **IIa**–**f** were determined against four fungal strains, including *A. flavus*, *A.* *niger*, *A. fumigatus*, *and G. candidum*, using clotrimazole as a reference standard. The antifungal activity assays were conducted according to the NCCLS standard M27-A method [28]. The MIC data of the tested compounds are presented in Table 2.

**Table 2:** MIC data (µg/mL) of the tested compounds against selected fungal strains.

Compound	MIC (µg/mL)			
	*A. flavus*	*A. niger*	*A. fumigatus*	*G. candidum*
**Ia**	32	32	32	32
**Ib**	16	16	8	32
**Ic**	32	>64	>64	>64
**Id**	>64	>64	32	>64
**Ie**	16	16	8	32
**If**	2	2	2	4
**IIa**	>64	>64	>64	>64
**IIb**	16	16	8	32
**IIc**	16	32	8	16
**IId**	>64	>64	>64	>64
**IIe**	32	>64	>64	32
**IIf**	4	4	4	8
**3**	32	>64	>64	>64
**(−)-Menthol**	32	32	32	>64
**Clotrimazole**	8	8	4	16

Negative control 5% DMSO---no activity.

The *in vitro* antifungal activity results showed that compounds **If** and **IIf** exhibited potent activities against all of the microorganisms tested. Furthermore, both of these compounds, especially **If**, exhibited activities comparable to those of the standard fungicide, clotrimazole, indicating that these compounds are worthy of further investigation. It was also found that compounds **Ib**, **Ie**, **IIb** and **IIc** showed potent *in vitro* antifungal activities, and that their activities were superior to those of the corresponding 3-(2-furyl)acrylic acid (**3**) and (−)-menthol. The other monosaccharide esters **Ia**, **Ic**, **Id**, **IIa**, **IId** and **IIe** showed no activity against the antifungal strains tested.

## 3. Experimental

### 3.1. Materials and Reagents

Melting points were determined in open glass capillaries using a paraffin bath and are uncorrected. ^1^H- and ^13^C-NMR spectra were measured on a Varian INOVA-400 instrument at 400 and 100 MHz, respectively, using TMS as an internal standard in CDCl_3_ or D_2_O solvents. IR spectra were obtained on a Thermo Nicolet AVATAR 370 FT-IR instrument using KBr plates. Mass spectra were recorded on a Waters Q-TOF Premier mass spectrometer. Optical rotation data were collected after mutarotation equilibration in about 10 min on a Perkin-Elmer 341 Polarimeter using HPLC grade anhydrous MeOH. All commercially available reagents and anhydrous solvents were purchased at the highest commercial quality and were used without further purification. The bacterial and fungal strains were obtained from Sichuan Industrial Institute of Antibiotics, Sinopharm Group Co. Ltd., (SINOPHARM-SIIA, Chengdu, China).

### 3.2. Chemical Synthesis

#### 3.2.1. Preparation of 3-(2-furyl)acrylic acid (**3**)

Furfural (28.8 g, 0.30 mol) was dissolved in AcOH (120 mL) and morpholine (26.1 g, 0.30 mol) was slowly added to the stirred mixture at 25 °C over a period of 10 min. Malonic acid (31.2 g, 0.30 mol) was then added to the reaction mixture and the resulting mixture was heated at reflux for 5.0 h. The mixture was then cooled to 25 °C and poured into cold water (400 mL). The resulting precipitate was collected by filtration, washed with water, and dried under vacuum at 40 °C in a vacuum oven to give the desired product. Yield: 35.2 g (85%), mp 140–141 °C (lit. m.p. 140–141 °C [24]).

#### 3.2.2. Preparation of 3-(2-furyl)acryloyl chloride (**4**)

Thionyl chloride (17.85 g, 0.15 mol) was added to a stirred solution of 3-(2-furyl)acrylic acid (**3**, 13.8 g, 0.1 mol) in CH_2_Cl_2_ (70 mL) at room temperature and the resulting mixture was heated at reflux for 3 h. The mixture was then cooled and concentrated *in vacuo* to afford 3-(2-furyl)acryloyl chloride quantitatively, which was used directly without further purification in the following reaction.

#### 3.2.3. A general procedure for the synthesis of compounds **Ia–f**

A solution of 3-(2-furyl)acryloyl chloride (**4**, 0.024 mol or 0.048 mol in the synthesis of **6f**) in dry CH_2_Cl_2_ (15 mL) was added in a dropwise manner to a solution of carbohydrate-acetonide **5a**–**f** (only one anomer was used, 0.02 mol) and Et_3_N (0.05 mol) in dry CH_2_Cl_2_ (60 mL) at 0 °C in an ice bath. The resulting solution was allowed to warm to room temperature and stirred for 12–15 h. The reaction mixture was then diluted with CH_2_Cl_2_ (40 mL) and washed sequentially with H_2_O (2 × 25 mL), saturated aqueous sodium bicarbonate (25 mL) and saturated aqueous sodium chloride (25 mL) and dried (Na_2_SO_4_). The solvent was then removed *in vacuo* and to give the crude products **6a**–**f** (only one anomer was obtained), which were used directly in the next steps without further purification. A 50% TFA-H_2_O solution (30 mL) was added to each of the compounds **6a**–**f** and the mixture was continuously stirred at room temperature for 3–5 h. Upon completion of the reaction (TLC, developing solvent: CHCl_3_/CH_3_OH = 15:1), the solvents were removed *in vacuo* and residue was purified by silica gel flash column chromatography (eluent: CHCl_3_/CH_3_OH = 10:1) to give the compounds **Ia**–**f** as light yellow foams.

*3-O-[3-(2-Furyl)acryloyl]-α/β-D-glucopyranose* (**Ia**): yield 48.5%, (α:β isomer = 2:3 based on ^1^H-NMR); [*α*]_D_^20^= +30.1 (c = 0.5, CH_3_OH); IR (KBr, cm^−1^): 3405.65, 2936.51, 1688.63, 1635.15, 1390.73, 1312.25, 1282.29, 1264.77, 1211.67, 1174.19, 1080.92, 1020.63, 751.60; ^1^H-NMR (D_2_O): *δ* 7.71 (s, 1H, Ar-H), 7.65 (d, *J* = 16 Hz, 1H, =CH), 6.90 (s, 1H, Ar-H), 6.66 (s, 1H, Ar-H), 6.46 (d, *J* = 16 Hz, 1H, =CH), 5.34 (d, *J* = 4.0 Hz, 0.4H, 1'-H_α_), 5.31 (d, *J* = 10.0 Hz, 0.6H, 1'-H_β_), 5.13 (t, *J* = 9.6 Hz, 0.6H, 3'-H_β_), 4.82 (t, *J* = 8.6 Hz, 0.6H, 3'-H_α_), 4.00–3.92 (m, 1H, 2'-H), 3.89–3.78 (m, 2H, 6'-H), 3.75–3.70 (m, 1H, 5'-H), 3.66–3.50 (m, 1H, 4'-H); ^13^C-NMR (D_2_O): *δ* 166.49, 166.30, 147.86, 143.49, 130.44, 130.33, 114.13, 111.38, 111.24, 110.27, 93.36, 89.59, 74.93, 73.26, 72.85, 70.03, 69.97, 68.74, 67.43, 65.47, 58.07, 57.90; ESI-MS (*m/z*): 623.5 [2M+Na]^+^, 339.05 [M+K]^+^, 323.10 [M+Na]^+^.

*6-O-[3-(2-Furyl)acryloyl]-α/β-D-galactopyranose* (**Ib**): yield 65.5%, (α:β isomer = 1:3 based on ^1^H-NMR); [*α*]_D_^20^= +33.2 (c = 0.5, CH_3_OH); IR (KBr, cm^−1^): 3421.48, 2921.46, 1689.56, 1646.43, 1484.23, 1311.05, 1287.52, 1267.55, 1219.03, 1178.91, 1068.70, 1028.97, 766.04; ^1^H-NMR (D_2_O): *δ* 7.70 (s, 1H, Ar-H), 7.60 (d, *J* = 16 Hz, 1H, =CH), 6.90 (s, 1H, Ar-H), 6.65 (s, 1H, Ar-H), 6.42 (d, *J* = 16 Hz, 1H, =CH), 5.33 (d, *J* = 4.0 Hz, 0.33H, 1'-H_α_), 4.65 (d, *J* = 8.0 Hz, 0.67H, 1'-H_β_), 4.47–4.36 (m, 2H, 2'-H, 3'-H), 4.10–4.01 (m, 2H, 6'-H), 3.95–3.86 (m, 1H, 5'-H), 3.73–3.54 (m, 1H, 4'-H); ^13^C-NMR (D_2_O): *δ* 171.05, 152.68, 148.31, 135.04, 118.96, 116.04, 115.16, 98.90, 94.83, 78.0, 74.97, 74.90, 74.12, 71.40, 71.19, 70.66, 66.23, 63.2, 62.5; ESI-MS (*m/z*): 623.18 [2M+Na]^+^, 339.06 [M+K]^+^, 323.10 [M+Na]^+^.

*1-O-[3-(2-Furyl)acryloyl]-β-D-fructopyranose* (**Ic**): yield 51.5%; [*α*]_D_^20^= −16.8 (c = 0.5, CH_3_OH); IR (KBr, cm^−1^): 3425.36, 2919.49, 1689.56, 1633.87, 1392.49, 1309.13, 1280.21, 1268.82, 1212.08, 1165.22, 1079.39, 751.26; ^1^H-NMR (D_2_O): *δ* 7.69 (s, 1H, Ar-H), 7.62 (d, *J* = 16 Hz, 1H, =CH), 6.90 (s, 1H, Ar-H), 6.64 (s, 1H, Ar-H), 6.43 (d, *J* = 16 Hz, 1H, =CH), 4.35(d, *J* = 11.6 Hz, 1H, 1'-H), 4.33 (d, *J* = 11.6 Hz, 1H, 1'-H), 4.12–4.06 (m, 2H, 4'-H+3'-H), 3.99–3.96 (m, 1H, 5'-H), 3.88–3.86 (m, 1H, 6'-H), 3.77–3.74 (m, 1H, 6'-H); ^13^C-NMR (D_2_O): *δ* 171.12, 152.79, 148.44, 135.40, 119.14, 115.94, 115.20, 99.62, 71.83, 71.46, 70.51, 68.19, 66.10; ESI-MS (*m/z*): 623.58 [2M+Na]^+^, 339.28 [M+K]^+^, 323.34 [M+Na]^+^.

*3-O-[3-(2-Furyl)acryloyl]-β-D-fructopyranose* (**Id**): yield 42.2%; [*α*]_D_^20^= −15.0 (c = 0.5, CH_3_OH); IR (KBr, cm^−1^): 3423.50, 2919.06, 1690.32, 1639.41, 1390.12, 1311.75, 1285.37, 1267.76, 1216.13, 1175.78, 1075.620, 1048.93, 750.55; ^1^H-NMR (D_2_O): *δ* 7.71 (s, 1H, Ar-H), 7.66 (d, *J* = 16 Hz, 1H, =CH), 6.92 (s, 1H, Ar-H), 6.65 (s, 1H, Ar-H), 6.42 (d, *J* = 16 Hz, 1H, =CH), 5.34 (d, *J* = 10.0 Hz, 1H, 3'-H), 4.18 (d, *J* = 10.8 Hz, 2H, 6'-H), 4.13 (s, 2H, 1'-H), 3.91–3.78 (m, 1H, 4'-H), 3.65–3.49 (m, 1H, 3'-H); ^13^C-NMR (D_2_O): *δ* 170.93, 152.77, 148.52, 135.64, 119.29, 115.79, 115.22, 99.64, 72.23, 71.69, 70.63, 66.43, 65.99; ESI-MS (*m/z*): 339.28 [M+K]^+^, 323.34 [M+Na]^+^.

*5-O-[3-(2-Furyl)acryloyl]-α/β-D-xylofuranose* (**Ie**): yield 54.8%, (α:β isomer = 3:1 based on ^1^H-NMR); [*α*]_D_^20^= +15.6 (c = 0.5, CH_3_OH); IR (KBr, cm^−1^): 3418.32, 2921.25, 1695.67, 1482.55, 1348.08, 1304.12, 1266.78, 1209.46, 1068.42, 1033.91, 752.10; ^1^H-NMR (D_2_O): *δ* 7.69 (s, 1H, Ar-H), 7.63 (d, *J* = 16 Hz, 1H, =CH), 6.89 (s, 1H, Ar-H), 6.64 (s, 1H, Ar-H), 6.43 (d, *J* = 16 Hz, 1H, =CH), 5.29 (d, *J* = 2.8 Hz, 0.75H, 1'-H_α_), 5.26–5.03 (m, 1H, 4'-H), 4.74 (d, *J* = 8.0 Hz, 0.25H, 1'-H_β_), 4.06–3.86 (m, 2H, 5'-H), 3.84–3.80 (m, 1H, 3'-H), 3.53–3.45 (m, 1H, 2'-H); ^13^C-NMR (D_2_O): *δ* 169.35, 151.66, 147.22, 132.54, 116.26, 114.84, 112.45, 99.72, 96.68, 77.66, 77.05, 76.12, 75.39, 73.96, 73.80, 65.55, 63.23; ESI-MS (*m/z*): 563.48 [2M+Na]^+^, 309.30 [M+K]^+^, 293.28 [M+Na]^+^.

*3,5-Bi-O-[3-(2-furyl)acryloyl]-α/β-D-xylofuranose* (**If**): yield 63.6%, (α:β isomer = 6:1 based on ^1^H-NMR); [*α*]_D_^20^= −15.4 (c = 0.5, CH_3_OH); IR (KBr, cm^−1^): 3417.95, 2921.02, 1703.85, 1638.68, 1479.26, 1351.47, 1308.86, 1264.24, 1210.32, 1168.12, 1077.95, 1045.66, 748.99; ^1^H-NMR (CDCl_3_): *δ* 7.50 (d, *J* = 2.0 Hz, 1H, Ar-H), 7.47 (d, *J* = 1.6 Hz, 1H, Ar-H), 7.44 (d, *J* = 16.0 Hz, 1H, =CH), 7.43 (d, *J* = 16.0 Hz, 1H, =CH), 6.65 (d, *J* = 3.2 Hz, 1H, Ar-H), 6.61 (d, *J* = 3.2 Hz, 1H, Ar-H), 6.48 (dd, *J* = 3.2, 1.6 Hz, 1H, Ar-H), 6.46 (dd, *J* = 3.2, 2.0 Hz, 1H, Ar-H), 6.32 (d, *J* = 16.0 Hz, 1H, =CH), 6.30 (d, *J* = 16.0 Hz, 1H, =CH), 5.57 (d, *J* = 4.4 Hz, 0.85H, 1'-H_α_), 5.37 (d, *J* = 8.4 Hz, 0.15H, 1'-H_β_), 5.35–5.28 (m, 1H, 2'-H), 4.76–4.70 (m, 1H, 4'-H), 4.40–4.35 (m, 2H, 5'-H), 4.34–4.29 (m, 1H, 3'-H); ^13^C-NMR (CDCl_3_): *δ* 166.84, 166.75, 150.66, 150.44, 145.27, 144.92, 132.69, 132.61, 131.88, 131.78, 115.98, 115.30, 115.24, 114.86, 113.97, 112.45, 112.29, 102.98, 96.08, 78.66, 77.35, 77.03, 76.72, 75.39, 74.98, 63.80, 62.47; ESI-MS (*m/z*): 803.25 [2M+Na]^+^, 429.12 [M+K]^+^, 413.14 [M+Na]^+^.

#### 3.2.4. Preparation of Menthyl Chloroformate **8**

A solution of pyridine (21.9 mL, 0.27 mmol) in toluene (150 mL) was added in a dropwise manner to a stirred solution of triphosgene (21.96 g, 0.074 mol) in toluene (260 mL) at 0 °C under an argon atmosphere. Stirring was continued for 15 min at 0 °C and a solution of (−)-menthol (**7**, 28.12 g, 0.18 mol) in toluene (100 mL) was then slowly added through a dropping funnel. The reaction mixture was allowed to warm to ambient temperature and stirred for 15 h. The reaction mixture was then diluted with water (300 mL) and extracted with toluene (2 × 200 mL). The combined organics were washed sequentially with water (200 mL) and brine (200 mL) and dried (Na_2_SO_4_). The solvent was removed *in vacuo* to give the title compound **8** as colorless oil (39.3 g, quant.), which was used directly in the next step without further purification.

#### 3.2.5. A General Procedure for the Synthesis of Compounds **IIa**–**f**

A solution of menthyl chloroformate **8** (0.024 mol or 0.05 mol for the synthesis of **9f**) in dry CH_2_Cl_2_ (15 mL) was added in a drop-wise manner to a solution of carbohydrate-acetonide **5a**–**f** (only one anomer was used, 0.02 mol) and Et_3_N (0.05 mol) in dry CH_2_Cl_2_ (60 mL) at 0 °C in an ice bath. The resulting solution was allowed to warm to room temperature and stirred for 12–15 h. The reaction mixture was then diluted with CH_2_Cl_2_ (40 mL) and washed sequentially with H_2_O (2 × 25 mL), saturated aqueous sodium bicarbonate (25 mL) and saturated aqueous sodium chloride (25 mL) before being dried (Na_2_SO_4_). The solvent was removed *in vacuo* to give a crude product from **9a**–**f** (only one anomer was obtained**)**, which was used directly in the next step without further purification. A 50% TFA-H_2_O solution (30 mL) was added to one of the compounds **9a**–**f** and the resulting mixture continuously stirred at room temperature for 3–5 h. Upon completion of the reaction (TLC, developing solvent: CHCl_3_/CH_3_OH = 20:1), the solvents were removed *in vacuo* and the crude residue was purified by silica gel flash column chromatography (eluent: CHCl_3_/CH_3_OH = 30:1) to give the compounds **IIa**–**f** as a colorless foam.

*3-O-Menthyloxycarbonyl-α/β-D-glucopyranose* (**IIa)**: yield 60.0%, (α:β isomer = 1:1 based on ^1^H-NMR); [*α*]_D_^20^ = +5.6 (c = 1.0, CH_3_OH); ^1^H-NMR (CDCl_3_): *δ* 5.36–5.30 (m, 0.5H, 1'-H_β_), 5.00–4.90 (m, 0.5H, 1'-H_α_), 4.82–4.72 (m, 1H, 3'-H), 4.58–4.48 (m, 1H, 5'-H), 4.05–3.92 (m, 2H, 4'-H+CHO), 3.83–3.72 (m, 1H, 6'-H), 3.70–3.44 (m, 2H, 6'-H+2'-H), 2.10–2.02 (m, 1H, CH), 2.01–1.92 (m, 1H, CH), 1.72–1.62 (m, 2H, CH_2_), 1.54–1.38 (m, 2H, CH+CH_2_), 1.12–0.99 (m, 2H, CH_2_), 0.91 (d, *J* = 6.4 Hz, 3H, CH_3_), 0.88 (d, *J* = 6.4 Hz, 3H, CH_3_), 0.88–0.84 (m, 1H, CH_2_), 0.76 (d, *J* = 4.4 Hz, 3H, CH_3_); ^13^C-NMR (CDCl_3_): *δ* 156.10, 92.30, 88.45, 79.52, 73.55, 73.08, 72.80, 71.05, 70.66, 69.25, 68.82, 62.02, 58.40, 57.96, 47.02, 40.48, 34.02, 31.35, 25.94, 23.24, 22.00, 20.68, 16.21; ESI-MS (*m/z*): 401.28 [M+K]^+^, 385.30 [M+Na]^+^.

*6-O-Menthyloxycarbonyl-α/β-D-galactopyranose* (**IIb**): yield 65.0%, (α:β isomer = 1:4 based on ^1^H-NMR), [*α*]_D_^20^ = −9.9 (c = 1.0, CH_3_OH); ^1^H-NMR (CDCl_3_): *δ* 5.38 (d, *J* = 3.6 Hz, 0.2H, 1'-H_α_), 4.64 (d, *J* = 8.4 Hz, 0.8H, 1'-H_β_), 4.63–4.47 (m, 1H, 6'-H), 4.38–4.06 (m, 2H, 2'-H+6'-H), 4.05–3.95 (m, 2H, 3'-H+CHO), 3.82–3.75 (m, 1H, 5'-H), 3.73–3.62 (m, 1H, 4'-H), 2.07–2.04 (m, 1H, CH), 2.01–1.92 (m, 1H, CH), 1.72–1.63 (m, 2H, CH_2_), 1.52–1.34 (m, 2H, CH+CH_2_), 1.10–0.99 (m, 2H, CH_2_), 0.91 (d, *J* = 6.0 Hz, 3H, CH_3_), 0.88 (d, *J* = 6.8 Hz, 3H, CH_3_), 0.86-0.84 (m, 1H, CH_2_), 0.76 (d, *J* = 6.8 Hz, 3H, CH_3_); ^13^C-NMR (CDCl_3_): *δ* 154.89, 96.88, 92.52, 78.97, 73.10, 72.74, 72.28, 69.60, 69.06, 68.78, 67.30, 66.90, 66.23, 66.02, 46.90, 40.60, 34.02, 31.34, 25.84, 23.14, 21.97, 20.76, 16.16; ESI-MS (*m/z*): 401.31 [M+K]^+^, 385.29 [M+Na]^+^.

*1-O-Menthyloxycarbonyl-β-D-fructopyranose* (**IIc**): yield 60.0%; [*α*]_D_^20^= −44.8 (c = 1.0, CH_3_OH); ^1^H-NMR (CDCl_3_): *δ* 4.54 (d, *J* = 11.2 Hz, 1H, 1'-H), 4.52 (d, *J* = 11.6 Hz, 1H, 1'-H), 4.34–4.25 (m, 2H, 4'-H+3'-H), 4.14–4.06 (m, 1H, 5'-H), 4.05–4.00 (m, 1H, CHO), 3.92–3.82 (m, 1H, 6'-H), 3.80–3.73 (m, 1H, 6'-H), 2.07–2.03 (m, 1H, CH), 2.00–1.90 (m, 1H, CH), 1.72–1.64 (m, 2H, CH_2_), 1.52–1.36 (m, 2H, CH+CH_2_), 1.10–0.99 (m, 2H, CH_2_), 0.91 (d, *J* = 7.2 Hz, 3H, CH_3_), 0.88 (d, *J* = 7.2 Hz, 3H, CH_3_), 0.86–0.84 (m, 1H, CH_2_), 0.76 (d, *J* = 7.2 Hz, 3H, CH_3_); ^13^C-NMR (CDCl_3_): *δ* 155.03, 96.81, 79.32, 73.39, 70.31, 69.25, 67.51, 63.57, 46.92, 40.54, 33.99, 31.34, 25.86, 23.13, 21.96, 20.73, 16.13; ESI-MS (*m/z*): 401.29 [M+K]^+^, 385.29 [M+Na]^+^.

*3-O-Menthyloxycarbonyl-β-D-fructopyranose* (**IId**): yield 53.4%; [*α*]_D_^20^= −61.3 (c = 1.0, CH_3_OH); ^1^H-NMR (CDCl_3_): *δ* 4.89 (d, *J* = 9.6 Hz, 1H, 3'-H), 4.59–4.51 (m, 1H, 6'-H), 4.15–4.10 (m, 1H, 6'-H), 4.06 (s, 2H, 1'-H), 3.88–3.82 (m, 1H, CHO), 3.67–3.63 (m, 1H, 4'-H), 3.52–3.45 (m, 1H, 3'-H), 2.10–2.03 (m, 1H, CH), 1.98–1.88 (m, 1H, CH), 1.70–1.67 (m, 2H, CH_2_), 1.54–1.36 (m, 2H, CH+CH_2_), 1.14–0.99 (m, 2H, CH_2_), 0.91 (d, *J* = 7.2 Hz, 3H, CH_3_), 0.88 (d, *J* = 7.2 Hz, 3H, CH_3_), 0.87–0.85 (m, 1H, CH_2_), 0.76 (d, *J* = 7.8 Hz, 3H, CH_3_); ^13^C-NMR (CDCl_3_): *δ* 155.67, 97.06, 79.59, 73.90, 69.79, 68.67, 64.74, 63.16, 46.90, 40.46, 33.97, 31.35, 26.12, 23.23, 21.95, 20.66, 16.18; ESI-MS (*m/z*): 401.30 [M+K]^+^, 385.31 [M+Na]^+^.

*5-O-Menthyloxycarbonyl-α/β-D-xylofuranose* (**IIe**): yield 42.0%, (α:β isomer = 4:1 based on ^1^H-NMR); [*α*]_D_^20^= −29.7 (c = 1.0, CH_3_OH); ^1^H-NMR (CDCl_3_): *δ*5.53 (d, *J* = 2.0 Hz, 0.8H, 1'-H_α_), 4.58–4.48 (m, 1.2H, 1'-H_β_+4'-H), 4.46–4.38 (m, 2H, 3'-H+5'-H), 4.36–4.24 (m, 2H, 2'-H+5'-H), 4.20–4.13 (m, 1H, CHO), 2.10–2.03 (m, 1H, CH), 1.98–1.90 (m, 1H, CH), 1.72–1.64 (m, 2H, CH_2_), 1.53–1.36 (m, 2H, CH+CH_2_), 1.10–0.99 (m, 2H, CH_2_), 0.92 (d, *J* = 7.2 Hz, 3H, CH_3_), 0.89 (d, *J* = 6.8 Hz, 3H, CH_3_), 0.87–0.85 (m, 1H, CH_2_), 0.77 (d, *J* = 6.8 Hz, 3H, CH_3_); ^13^C-NMR (CDCl_3_): δ 155.21, 155.06, 102.80, 96.09, 80.15, 79.83, 79.19, 79.10, 76.36, 75.70, 75.47, 69.85, 67.40, 66.45, 65.96, 46.83, 40.54, 33.94, 31.31, 25.86, 23.12, 21.92, 20.68, 16.13; ESI-MS (*m/z*): 371.27 [M+K]^+^, 355.31 [M+Na]^+^.

*3,5-Di-O-menthyloxycarbonyl-α/β-D-xylofuranose* (**IIf**): yield 31.5%, (α:β isomer = 3:1 based on ^1^H-NMR); [*α*]_D_^20^= −40.2 (c = 1.0, CH_3_OH); ^1^H-NMR (CDCl_3_): *δ*5.21 (d, *J* = 3.6 Hz, 0.75H, 1'-H_α_), 5.02 (d, *J* = 8.4 Hz, 0.25H, 1'-H_β_), 4.61–4.47 (m, 3H, 4'-H+5'-H+3'-H), 4.38–4.29 (m, 1H, 2'-H), 4.29–4.14 (m, 2H, 5'-H+CHO), 4.14–3.96 (m, 1H, CHO), 2.09-2.03 (m, 2H, 2CH), 1.98–1.91 (m, 2H, 2CH), 1.72–1.57 (m, 4H, 2CH_2_), 1.53–1.36 (m, 4H, 2CH+2CH_2_), 1.16–0.99 (m, 4H, 2CH_2_), 0.96–0.85 (m, 16H, 4CH_3_+2CH_2_), 0.84–0.75 (m, 6H, 2CH_3_); ^13^C-NMR (CDCl_3_) δ: 155.66, 155.62, 101.45, 96.35, 79.75, 79.60, 79.12, 78.03, 77.16, 76.58, 75.30, 73.67, 63.72, 63.25, 46.85, 40.58, 33.92, 31.38, 26.32, 23.20, 21.95, 20.67, 16.20; ESI-MS (*m/z*): 553.21 [M+K]^+^, 537.44 [M+Na]^+^, 493.49 [M-CO_2_+Na]^+^.

### 3.3. Antibacterial and Antifungal Activity Assays

For each tested compound, 1.28 mg sample was put in a 25 mL flask and dissolved in 10 mL 5% DMSO. The drug was filtered into autoclaving centrifuge tubes in super clean bench. Penicillin, streptomycin and clotrimazole solutions were prepared to the same concentration as the positive control drug. After all bacteria and fungi were recovered, each single colony was picked and inoculated in 3 mL of either a sterilized Mueller-Hinton (MH) broth cultured at 37 °C for 18–24 h (for bacteria) or a Sabouraud Dextrose (SD) broth cultured at 28 °C for 48 h (for fungi). The microorganism solution was corrected to 0.5 McFarland standard turbidity using either the MH or SD broths, and subsequently diluted by 1:100 (amount of microorganism approximately 10^6^ colony forming units/mL) with either the MH or SD broth and inoculated immediately. Blank broth (100 µL) was added into all the wells of rows 2–12 of a 96-well plate. Broth (180 µL) and dispensed drug liquor (20 µL) where added to the first row of the 96-well plate and the mixture was well mixed. A sample of the resulting mixture (100 µL) was inhaled and added into the corresponding wells of the second row. The same mixing and transferring operations were repeated until the wells in the 12th row were filled. The concentrations of drug in the wells of rows 1–12 were 128, 64, 32, 16, 8, 4, 2, 1, 0.5, 0.25, 0.125, 0.0625 µg/mL, respectively. The seventh and eighth rows of the 96-well plate were used for the positive and negative controls, respectively. A diluted microorganism solution (100 µL) was added to each well of the 96-well plate and the plate was shaken on a shaker instrument and subsequently stored in an incubator for 24–48 h at 37 °C (for bacteria) and at 28 °C (for fungi). Each experiment was performed in triplicate.

## 4. Conclusions

In summary, the design and synthesis of two novel series of 3-(2-furyl) acrylate monosaccharide esters **Ia**–**f** and menthyloxycarbonyl monosaccharide esters **IIa**–**f** from simple starting materials and under mild reaction conditions has been reported. The antibacterial and antifungal properties of these novel compounds were evaluated. The results revealed that the 3-(2-furyl)acrylate monosaccharide ester derivatives **Ia**–**f** were either inactive or only weakly active against the three Gram-positive bacterial strains tested (*B. subtilis*, *S. aureus*, *S.* *epidermidis*), whereas the menthyloxy carbonyl monosaccharide ester derivatives **IIa**–**f** exhibited greater degrees of activity, with compound **IIe** showing remarkably high antibacterial activity. Compound **Ib**, **Ie**, **IIb** and **IIc** displayed potent *in vitro* antifungal activities, whereas **If** and **IIf** showed promising antifungal activities against all of the microorganisms tested, with compound **If** exhibiting significantly high activities, which are worthy of further investigation.
